# Child–Parent Relationship Therapy for Earthquake‐Affected Preschoolers: A 1‐Year Controlled Study

**DOI:** 10.1002/cpp.70264

**Published:** 2026-04-10

**Authors:** Merve Nur Bozkurt Karali, Yasemin Özkan, Dilşad Foto Özdemir

**Affiliations:** ^1^ Hitit University Faculty of Health Sciences, Department of Social Work Çorum Türkiye; ^2^ Hacettepe University Faculty of Economics and Administrative Sciences, Department of Social Work Ankara Türkiye; ^3^ Hacettepe University Faculty of Medicine, Department of Child and Adolescent Psychiatry Ankara Türkiye

**Keywords:** child PTSD, child–parent relationship therapy, earthquake, mother PTSD, parent–child relationship

## Abstract

**Objective:**

This study examined the effectiveness of *Child–Parent Relationship Therapy* (CPRT) among earthquake‐affected preschool children and their mothers in Türkiye.

**Method:**

Using a quasi‐experimental controlled design, participants were assigned to experimental (*n* = 12), placebo (*n* = 12), and control (*n* = 12) groups. Parental empathy, parent–child relationship and children's posttraumatic emotional distress, affective and behavioural problems were assessed at pretest, posttest and 2‐, 6‐ and 12‐month follow‐ups.

**Results:**

Mothers in the CPRT group showed significant improvements in empathy. Children's loneliness/sleep problems decreased short term, while emotional distress and impulsivity declined significantly at 1‐year follow‐up. However, fear/anxiety scores increased across all groups, and no significant group differences were found in emotional–behavioural problems or parent–child relationship scores.

**Conclusion:**

CPRT emerged as an effective intervention in some aspects of children's posttrauma well‐being, yet ongoing environmental stressors and parents' psychological conditions limited the sustainability of therapeutic gains.

## Summary

- This 1‐year controlled study suggests that Child–Parent Relationship Therapy (CPRT) improves maternal empathy following earthquake exposure.
- CPRT was associated with reductions in children's posttraumatic emotional distress, impulsivity and loneliness/sleep difficulties that emerged during follow‐up, consistent with a sleeper‐effect pattern.
- Increases in children's fear and anxiety, together with limited change in children's emotional–behavioural problems and overall parent–child relationship, suggest that ongoing postdisaster stressors—such as recurrent aftershocks and parental psychological symptoms—may constrain recovery; however, CPRT appeared to buffer against declines in parent–child relationship quality observed in the control and placebo groups.
- Overall, CPRT appears to be a promising family‐based postdisaster intervention for preschool children, while findings highlight the importance of addressing parental mental health and ongoing disaster‐related stressors to support sustained child recovery.

## 1 Introduction

On 6 February 2023, two major earthquakes struck 11 provinces in Türkiye, causing immense destruction and loss of life. Beyond physical devastation, earthquakes create profound psychological and social impacts, with children representing the most vulnerable group (Hisli Şahin et al. 2011). Studies indicate that earthquake‐affected children often experience separation anxiety, sleep problems, clinging behaviour, nightmares, fear, anger and regression (Özer Yanarkaya and Dikici Sığırtmaç 2025; Kim 2018; Yorbik et al. 2004; Kowalski and Kalayjian 2001). Parents frequently reported that they experienced severe anxiety, insomnia, PTSD symptoms and difficulty coping with uncertainty (Kowalski and Kalayjian 2001; Tunç and Candemir 2025; Taner Derman and Türen 2025).

Primary caregivers play a critical role in helping young children process trauma due to their central position in the parent–child relationship (Scheeringa and Zeanah 2001). Exposure to parental stress directly affects children's emotional regulation (Anthony et al. 2005; Crnic et al. 2005; Huth‐Bocks and Hughes 2008). Therefore, family support represents a key protective factor in children's recovery. Accordingly, psychosocial interventions should target not only the child but the entire family (Cheng et al. 2018). Play‐based family interventions are recognized as effective tools for fostering postdisaster psychosocial recovery (Margolin et al. 2010; VanFleet and Sniscak 2003; Shen 2002).

Child–Parent Relationship Therapy (CPRT) (Landreth and Bratton 2019), an empirically supported model combining family and play therapy, effectively addresses children's posttrauma needs (Kim 2018; VanFleet and McCann 2007; VanFleet and Sniscak 2003). CPRT trains parents in non‐directive, child‐centered play, enabling them to conduct therapeutic play sessions and strengthen parent–child bonds (Guerney and Ryan 2013; Landreth and Bratton 2019). Studies conducted in trauma and disaster contexts demonstrate that CPRT and filial therapy–based interventions produce statistically significant and consistent effects. Following the Nepal earthquake, Kim (2018) reported that a 6‐session filial therapy intervention significantly reduced children's somatic symptoms (*z* = −3.09, *p* < 0.01) and improved parent–child interaction (*z* = −2.98, *p* < 0.01). Tal et al. (2018) found significant time effects following CPRT on children's internalizing and externalizing behaviours (*Wald =* 43.62 and 55.52; *p* < 0.001), as well as reductions in parental stress and secondary traumatization (*p* < 0.001). Similarly, Hanif and Gul (2023) reported a significant decrease in total behavioural problems among trauma‐exposed children (*t*(29) = 9.78, *p* < 0.001) with a moderate effect size (*d =* 0.57). In another study with earthquake‐affected children, filial therapy resulted in significant reductions across all dimensions of PTSD symptoms compared to the control group (*p* < 0.05) (Bahramian and Samsam Shariat 2024). Meta‐analytic findings further support these results: Bratton et al. (2005), in a meta‐analysis of 93 controlled studies, reported a large overall effect size for play therapy (*d =* 0.80), with stronger effects in parent‐involved (filial therapy) interventions (*d =* 1.15). Lin and Bratton (2015) found a significant overall effect for child‐centered play therapy (*effect size* = 0.47, *p* < 0.001) and stronger outcomes when caregivers were involved (*effect size* = 0.59; filial therapy and CPRT). More recently, Taylor (2024) reported that CPRT produced a large effect size (*g =* 1.04) and demonstrated superiority over no‐treatment control groups (*g =* 0.50). By empowering parents as therapeutic agents (VanFleet and Sniscak 2003), CPRT offers a valuable approach that may support parent and child well‐being in trauma‐affected families.

Although many studies have explored postdisaster interventions for children (de Roos et al. 2011; Giannopoulou et al. 2006; Lee and Jang 2020), few have involved parents as active therapeutic agents. Filial Therapy—an extended form of CPRT—has reduced PTSD and somatization symptoms in school‐age children following earthquakes (Bahramian and Samsam Shariat 2024; Kim 2018). However, controlled and longitudinal CPRT studies on preschoolers after earthquakes remain scarce. This study addresses this gap by directly involving parents, focusing on preschoolers and employing experimental, placebo and control groups with 2‐, 6‐ and 12‐month follow‐ups. The study aims to evaluate the effectiveness of CPRT on parent–child relationships, parental empathy and children's emotional and behavioural outcomes among mothers affected by the Kahramanmaraş earthquakes. To achieve this objective, the research was guided by the following questions:

- What are the sociodemographic characteristics of the participating children and parents?
- Do the experimental, placebo and control groups differ over time (pretest, posttest, 2, 6, 12 months) in parent–child relationship, parental empathy and children's emotional distress and emotional and behavioural problems?
- Within each group, do these outcomes change across the same time points?
- How are maternal depression, anxiety and PTSD related to these outcomes at pretest and at the 1 year follow‐up?

## 2 Methods

### 2.1 Participants

Participants were selected through purposive sampling and included mothers of children aged 3–6 years. Although CPRT is designed for ages 3–10 (Bratton and Landreth 2006), the study focused on 3–6 years because developmental responses to trauma vary markedly across this range. Mothers and children with diagnosed psychiatric disorders, injuries, entrapment under debris or loss of a first‐degree relative were excluded, as such cases require individualized therapy (VanFleet and Sniscak 2003). Only mothers participated because they are typically the primary caregivers of young children in Türkiye (Karabekiroglu et al. 2013).

Inclusion criteria were: residence in an area affected by the 6 February 2023 earthquakes; having a 3‐ to 6‐year‐old child who experienced the earthquake with the parent; internet access; and voluntary participation. Exclusion criteria were: living outside affected provinces, being a father, having a child outside the 3–6 age range, experiencing severe loss or injury, or having a pre‐existing psychiatric diagnosis. Based on these criteria, the final sample consisted of 36 mothers of 3‐ to 6‐year‐old children living in the earthquake‐affected region, who met all inclusion conditions and consented to participate.

Sample size was calculated using G*Power 3.1.9.7. A one‐way ANOVA with a power of 0.80, an effect size of 0.60, and an alpha level of 0.05 indicated a minimum of 10 participants per group. To offset attrition, the sample was expanded to 36 mothers (12 per experimental, control and placebo groups), randomly assigned by lot.

As shown in Table 1, most mothers were aged 23–35 years and held a bachelor's degree. Child gender was balanced in the experimental and placebo groups, while boys were more frequent in the control group (58.3%). Most children were 3–4 years old and had no diagnosed disorders.

**TABLE 1:** Sociodemographic characteristics of the groups.

		Experimental		Control		Placebo	
Variables	Group	*n*	%	*n*	%	*n*	%
Age	23–35	10	83.3	6	50.0	7	58.3
	36–47	2	16.7	6	50.0	5	41.7
Educational status	Secondary education	2	16.7	2	16.7	1	8.3
	Bachelor's degree	9	75.0	9	75.0	9	75.0
	Postgraduate	1	8.3	1	8.3	2	16.7
Child's gender	Girl	6	50.0	5	41.7	6	50.0
	Boy	6	50.0	7	58.3	6	50.0
Child's age	3–4	7	58.3	8	66.7	7	58.3
	5–6	5	41.7	4	33.3	5	41.7

Levene's test indicated homogeneity of variance (*p* > 0.05). At pretest, significant differences were found in Beck Anxiety Inventory (BAI; *F*(2,33) = 3.72, *p* = 0.035) and DSM‐5 Posttraumatic Stress Disorder Checklist (PCL‐5; *F*(2,33) = 5.31, *p* = 0.010) scores between control and placebo groups, but not with the experimental group. Because both groups were under non‐treatment conditions, these differences were not considered a methodological threat to the experimental design.

### 2.2 Procedure

This paper reports the quantitative arm of a mixed‐methods, quasi‐experimental project evaluating CPRT. We used a 3 × 3 factorial quasi‐experimental design (Group: experimental, placebo, control × Time: pretest, posttest, follow‐ups). The experimental group received CPRT; the placebo group received a researcher‐developed Earthquake Awareness Training; the control group received no intervention. In experimental research, participants' awareness of being involved in a study may lead to conscious or unconscious behavioural changes, a phenomenon referred to in the literature as the Hawthorne effect (Adair 1984; Kocakaya 2012). To reduce such validity threats, a placebo group was included as a third condition in the study. The placebo group received an Earthquake Awareness Training developed by the researchers, delivered once weekly over 10 sessions, similar in structure to CPRT. Although similar in structure, this training was deliberately designed to be entirely different from CPRT in terms of its theoretical foundations and intervention content.

The study received ethical approval and complied with the Declaration of Helsinki. After approval, participants provided informed consent; confidentiality and voluntary participation were ensured. Data collection began online in January 2024. At pretest, mothers completed the BDI, BAI, PCL‐5 and child‐focused measures (PEDS, PCRS, FPC), as described below. CPRT then commenced for the experimental group. Consistent with Bratton and Landreth's (2006) guidance for small‐group CPRT, 12 mothers were assigned to two online groups (*n* = 5 and *n* = 7).

CPRT met weekly for 2‐h sessions across approximately 2.5 months, for a total of 10 meetings. To mitigate participation barriers (Boswell 2014), sessions were delivered online; prior work supports the feasibility and effectiveness of online CPRT (Hicks and Baggerly 2017). The placebo group intervention was delivered concurrently in an online format.

Posttests were administered immediately after the intervention, and follow‐ups at 2 and 6 months (PEDS, PCRS, FPC only). At 1 year, all measures (BDI, BAI, PCL‐5, PEDS, PCRS, FPC) were repeated.

### 2.3 Measures

Data were collected using a socio‐demographic information form prepared by the researcher and the following scales:

*Parent–Child Relationship Scale* (PCRS; Hetherington and Clingempeel 1992; Turkish: Aytaç et al. 2018) assesses relationship quality with 15 items on a 5‐point Likert scale, yielding Positive and Negative subscales; reported reliability coefficients exceed 0.80. Validity was supported by the scale's two‐factor structure and acceptable composite reliability coefficients reported in both the original and Turkish adaptation studies (Aytaç et al. 2018).
*Filial Problem Checklist* (FPC; Turkish: Güler Öztekin et al. 2022) comprises 108 items measuring Problematic Behaviors, Emotional Control Difficulties, Insecurity/Negative Emotions and Problematic Social Behaviors. The Cronbach's alpha coefficient for the Turkish version was reported as 0.96. Validity was supported by factor analytic evidence and significant item–total correlations (Güler Öztekin et al. 2022).
*Measurement of Empathy in Adult‐Child Interaction* (MEACI; Guerney et al. 1968; Turkish: Güler Öztekin and Gülbahçe 2019) evaluates parents' skills acquired through CPRT during special play sessions across three dimensions: Scores range 18–90, with lower scores reflecting greater parental empathy. Validity was supported by the scale's theoretically grounded structure assessing empathic behaviour across three core dimensions and its expert‐based observational scoring system (Güler Öztekin and Gülbahçe 2019). Sessions were coded in 3‐min segments by two independent raters (researcher; CPRT‐experienced clinician), and mean ratings were analysed, establishing inter‐rater reliability (Güler Öztekin and Gülbahçe 2019; Ada 2015). Inter‐rater reliability was evaluated using intraclass correlation coefficients (ICC; two‐way mixed‐effects model, absolute agreement). For the MEACI total score, reliability was excellent at both pretest (ICC = 0.988) and posttest (ICC = 0.856).
*Pediatric Emotional Distress Scale* (PEDS; Saylor et al. 1999; Turkish: Göktepe 2014) evaluates posttraumatic stress symptoms in children aged 2–10 years. It consists of 21 items across five subscales: Impulsivity, Fear/Anxiety, Loneliness/Sleep Problems, Attention/Memory Problems and Somatization/Regression. The internal consistency reliability was reported as 0.85. Validity was supported by the scale's five‐factor structure identified through exploratory factor analysis, item discrimination indices and evidence of convergent validity reported in the Turkish adaptation study (Göktepe 2014).
*Posttraumatic Stress Disorder Checklist for DSM‐5* (PCL‐5; Weathers et al. 2013; Turkish: Boysan et al. 2017) consists of 20 items and four subscales, with the composite reliability coefficients of Re‐experiencing (0.79–0.92), Avoidance (0.73–0.91), Negative Alterations (0.85–0.90), and Hyper‐arousal (0.81–0.88). The PCL‐5 provides a diagnostic cutoff of ≥ 47 for PTSD. Validity was supported by a four‐factor structure confirmed through confirmatory factor analysis and strong associations with related trauma‐, anxiety‐ and depression‐related measures (Boysan et al. 2017).
*Beck Anxiety Inventory* (BAI; Beck et al. 1988; Turkish: Ulusoy et al. 1998) includes 21 items (*α* = 0.93) and two factors—Subjective Anxiety and Somatic Symptoms; conventional cutoffs are 8–15 (*mild*), 16–25 (*moderate*) and 26–63 (*severe*) (Beck and Steer 1993). Validity was supported by a two‐factor structure identified through factor analysis and significant correlations with related anxiety and depression measures (Ulusoy et al. 1998).
*Beck Depression Inventory* (BDI; Beck et al. 1961; Turkish: Hisli Sahin 1988/Hisli Şahin 1989) comprises 21 items, with reported reliability *r* = 0.74–0.80; the Turkish cutoff is 17, with scores ≥ 17 indicating clinically elevated depressive symptoms. (Hisli Sahin, 1988). Validity was supported by significant correlations between BDI scores and MMPI‐D depression scores in a psychiatric sample (Hisli Sahin 1988).

### 2.4 Data analysis

Data were analysed using SPSS version 27. Frequency tables were generated for sociodemographic variables. Normality assumption was tested with the Shapiro–Wilk test, reliability with Cronbach's alpha coefficients, and homogeneity of variance with Levene's test. When assumptions were met, between‐group differences were evaluated using one‐way analysis of variance (ANOVA) and post hoc tests. To compare group means, the Mann–Whitney *U* test was used for two groups, and the Kruskal–Wallis *H* test for three or more groups. Relationships between scales were examined using Spearman's correlation, and the effects of mothers' psychological symptoms on children's variables were tested through multiple regression analysis. Across all multiple regression models, no multicollinearity issues were detected among the independent variables (VIF < 10; Tolerance > 0.20). All analyses were conducted at a significance level of 0.05.

## 3 Results

In presenting the findings, the analyses followed a hierarchical structure. First, Table 2 provides a descriptive profile of maternal and child symptom levels at pretest and at the 1‐year follow‐up, without implying statistical comparison. Subsequently, correlation and regression analyses examined the associations and predictive role of maternal psychological symptoms on child outcomes (Tables 3, 4, 5, 6). Finally, Wilcoxon Signed‐Rank Tests evaluated within‐group changes over time and intervention‐related effects (Tables 7, 8, 9, 10, 11, 12, 13).

**TABLE 2:** Pretest and 1‐year follow‐up scale scores by group.

		Pretest			1‐year follow‐up		
Scale	Group	*N*	X̄	ss	*N*	X̄	ss
BAI	Experimental	12	21.00	13.83	12	15.67	10.07
	Placebo	12	26.08	12.99	10	9.00	10.43
	Control	12	12.25	10.70	10	7.40	5.38
BDI	Experimental	12	15.33	4.19	12	12.08	7.15
	Placebo	12	17.17	8.43	10	10.40	9.05
	Control	12	11.83	6.95	10	9.80	7.69
PCL‐5	Experimental	12	36.00	11.85	12	29.00	20.21
	Placebo	12	42.67	16.51	10	21.40	18.09
	Control	12	25.42	10.00	10	18.00	18.68
PEDS	Experimental	12	35.75	9.29	12	27.67	6.72
	Placebo	12	35.25	13.77	10	29.30	6.43
	Control	12	30.00	8.20	10	27.20	6.99
FPC	Experimental	12	46.08	36.39	12	43.25	41.75
	Placebo	12	47.17	76.12	10	37.70	36.39
	Control	12	38.17	46.48	10	25.70	32.73
PCRS (positive)	Experimental	12	41.92	3.66	12	41.58	4.34
	Placebo	12	39.83	6.83	10	35.20	10.39
	Control	12	43.08	3.87	10	44.10	3.21
PCRS (negative)	Experimental	12	15.33	4.89	12	14.67	5.09
	Placebo	12	13.00	3.16	10	12.90	4.46
	Control	12	13.08	3.40	10	16.20	3.58

Abbreviations: BAI = Beck Anxiety Inventory; BDI = Beck Depression Inventory; FPC = Filial Problem Checklist; PCL‐5 = PTSD Checklist for DSM‐5; PEDS = Pediatric Emotional Distress Scale; PCRS (Negative) = Parent–Child Relationship Scale—Negative Relationship subscale; PCRS (Positive) = Parent–Child Relationship Scale – Positive Relationship subscale.

**TABLE 3:** Predictive effects of mothers' psychological symptoms on PEDS scores (pretest).

Independent variables	B	SE	Standardized *β*	*t*	*p*	Tolerance	VIF
Constant	19,874	3.965	—	5.012	< 0.001	—	—
BAI	0.385	0.164	0.486	2.352	0.025	0.372	2.690
BDI	−0.081	0.243	−0.053	−0.335	0.739	0.645	1.551
PCL‐5	0.213	0.144	0.290	1.475	0.150	0.412	2.426

*Note:* *F*(3,32) = 10,310, *p* < 0.001, adjusted *R* ^2^ = 0.444.

**TABLE 4:** Predictive effects of mothers' psychological symptoms on positive parent–child relationship (pretest).

Independent variables	B	SE	Standardized *β*	*T*	*p*	Tolerance	VIF
Constant	45,774	2.319	—	19,739	< 0.001	—	—
BAI	−0.116	0.096	−0.310	−1.209	0.236	0.372	2.690
BDI	−0.209	0.142	−0.287	−1.473	0.151	0.645	1.551
PCL‐5	0.035	0.084	0.101	0.416	0.680	0.412	2.426

*Note:* *F*(3,32) = 2.924, *p* = 0.049, adjusted *R* ^2^ = 0.142.

**TABLE 5:** Predictive effects of mothers' psychological symptoms on peds scores (1‐year follow‐up).

Independent variables	B	SE	Standardized *β*	*T*	*p*	Tolerance	VIF
Constant	20,886	1.342	—	15,560	< 0.001	—	—
BAI	0.075	0.102	0.109	0.737	0.467	0.615	1.625
BDI	0.240	0.197	0.284	1.216	0.234	0.245	4.085
PCL‐5	0.159	0.085	0.459	1.866	0.073	0.220	4.541

*Note:* *F*(3,28) = 15,631, *p* < 0.001, adjusted *R* ^2^ = 0.586.

**TABLE 6:** Predictive effects of mothers' psychological symptoms on children's fpc scores (1‐year follow‐up).

Independent variables	B	SE	Standardized *β*	*T*	*p*	Tolerance	VIF
Constant	12,264	8.598	—	1.426	0.165	—	—
BAI	−0.502	0.653	−0.128	−0.769	0.449	0.615	1.625
BDI	−2.099	1.263	−0.441	−1.662	0.108	0.245	4.085
PCL‐5	2.218	0.544	1.139	4.075	< 0.001	0.220	4.541

*Note:* *F*(3,28) = 10,048, *p* < 0.001, adjusted *R* ^2^ = 0.467.

**TABLE 7:** Wilcoxon signed‐rank test results for MEACI.

Subscale	Time	*N*	Mean (SD)	*z*	*p*
Acceptance of the child	Posttest	12	7.75 (2.98)	−3.071	0.002*
	Pretest	12	17.00 (7.27)		
Allowing the child to lead	Posttest	12	7.75 (2.98)	−2.949	0.003*
	Pretest	12	19.50 (7.83)		
Involvement in play	Posttest	12	6.25 (0.86)	−2.524	0.012*
	Pretest	12	15.00 (9.04)		
MEACI total	Posttest	12	21.75 (5.29)	−3.061	0.002*
	Pretest	12	51.08 (21.53)		

* *p* < 0.05.

**TABLE 8:** Wilcoxon signed‐rank test results for PEDS subscale: Loneliness/sleep.

Time 1–time 2 comparison	Group	*n*	Time 1 mean (SD)	Time 2 mean (SD)	*z*	*p*
Pretest–2 months	Experimental	12	6.83 (2.04)	5.50 (2.02)	−2.064	0.039*
Pretest–6 months	Control	9	6.56 (1.88)	5.22 (2.17)	−2.414	0.016*
Pretest–1 year	Experimental	12	6.83 (2.04)	5.08 (1.44)	−2.319	0.020*
Pretest–1 year	Control	9	6.44 (2.00)	4.67 (2.30)	−2.309	0.021*
Pretest–1 year	Placebo	11	7.18 (2.60)	5.55 (1.81)	−2.200	0.028*
Posttest–1 year	Experimental	12	6.58 (2.39)	5.08 (1.44)	−2.203	0.028*
2‐month–1 year	Control	8	6.00 (2.61)	4.88 (2.36)	−2.165	0.030*
6‐month–1 year	Experimental	12	5.92 (1.44)	5.08 (1.44)	−2.126	0.033*

* *p* < 0.05.

**TABLE 9:** Wilcoxon signed‐rank test results for PEDS subscale: Impulsivity.

Time 1–time 2 comparison	Group	*n*	Time 1 mean (SD)	Time 2 mean (SD)	*z*	*p*
Pretest–1 year	Experimental	12	9.58 (3.03)	8.00 (2.76)	−2.111	. 035*
2 months–6 months	Placebo	12	8.75 (2.77)	7.42 (2.50)	−2.585	0.010*
2 months–1 year	Experimental	12	8.92 (2.97)	8.00 (2.76)	−2.157	0.031*
2 months–1 year	Placebo	11	8.82 (2.89)	7.73 (2.72)	−2.041	0.041*

* *p* < 0.05.

**TABLE 10:** Wilcoxon signed‐rank test results for PEDS subscale: Fear/anxiety.

Time 1–time 2 comparison	Group	*n*	Time 1 mean (SD)	Time 2 mean (SD)	*z*	*p*
Pretest–posttest	Experimental	12	3.00 (1.04)	3.92 (1.17)	−2.495	0.013*
Pretest–posttest	Control	11	2.73 (1.01)	4.91 (1.70)	−2.961	0.003*
Pretest–2 months	Experimental	12	3.00 (1.04)	4.08 (1.62)	−2.588	0.010*
Pretest–2 months	Control	9	2.78 (1.09)	4.78 (2.17)	−2.555	0.011*
Pretest–6 months	Experimental	12	3.00 (1.04)	4.25 (1.42)	−2.683	0.007*
Pretest–6 months	Control	9	2.78 (1.09)	4.44 (2.13)	−2.807	0.005*
Pretest–1 year	Experimental	12	3.00 (1.04)	3.75 (1.06)	−2.714	0.007*
Pretest–1 year	Control	9	2.78 (1.09)	4.78 (1.87)	−2.699	0.007*
Posttest–6 months	Placebo	12	4.50 (1.31)	3.50 (0.67)	−2.136	0.033*
6 months–1 year	Placebo	11	3.55 (0.69)	4.73 (1.27)	−2.232	0.026*

* *p* < 0.05.

**TABLE 11:** Wilcoxon signed‐rank test results for PEDS total score.

Time 1–time 2 comparison	Group	*N*	Time 1 mean (SD)	Time 2 mean (SD)	*z*	*p*
Pretest–2 months	Experimental	12	26.92 (7.62)	23.67 (7.17)	−1.962	0.051
Pretest–6 months	Placebo	12	29.25 (10.63)	21.67 (5.09)	−2.268	0.023*
Pretest–1 year	Experimental	12	26.92 (7.62)	22.17 (5.92)	−2.485	0.013*
Pretest–1 year	Placebo	11	29.91 (10.89)	23.27 (5.82)	−2.100	0.036*
Posttest–1 year	Experimental	12	24.58 (5.90)	22.17 (5.92)	−2.011	0.044*
Posttest–1 year	Placebo	11	24.64 (6.31)	23.27 (5.82)	−1.973	0.049*
6 Months–1 year	Experimental	12	24.08 (5.99)	22.17 (5.92)	−2.469	0.014*

* *p* < 0.05.

**TABLE 12:** Wilcoxon signed‐rank test results for FPC (subscales and total score).

Subscale	Time 1–time 2 comparison	Group	*N*	Time 1 mean (SD)	Time 2 mean (SD)	*z*	*p*
Emotional Control Difficulties	Posttest–6 months	Control	9	13.67 (17.727)	11.22 (17.541)	−2.132	0.033*
	Posttest–1 year	Placebo	11	20.00 (12.892)	14.09 (12.942)	−2.092	0.036*
Problematic Social Behaviors	Pretest–6 months	Control	9	4.00 (6.205)	2.67 (4.899)	−2.232	0.026*
	Posttest–1 year	Control	9	4.78 (5.630)	1.78 (3.153)	−1.997	0.046*
	2 months–1 year	Control	8	4.50 (4.536)	1.75 (3.370)	−2.226	0.026*
Insecurity and Negative Emotions	Pretest–6 months	Control	9	14.22 (14.131)	10.67 (14.177)	−2.032	0.042*
	Posttest–6 months	Control	9	13.00 (15.116)	10.67 (14.177)	−2.214	0.027*
	Pretest–1 year	Control	9	14.78 (13.818)	8.11 (10.948)	−2.524	0.012*
	Posttest–1 year	Placebo	11	13.18 (9.908)	9.45 (8.583)	−2.077	0.038*
Filial Problem Checklist Total	Posttest–6 months	Control	9	36.89 (46.745)	30.44 (45.092)	−2.325	0.020*
	Posttest–1 year	Placebo	11	48.73 (31.563)	36.91 (37.774)	−2.040	0.041*

* *p* < 0.05.

**TABLE 13:** Wilcoxon signed‐rank test results for *PCRS* (positive and negative subscale).

Subscale	Time 1–time 2 comparison	Group	*n*	Time 1 mean (SD)	Time 2 mean (SD)	*z*	*p*
*PCRS* (positive)	Pretest–2 months	Control	9	45.22 (2.819)	40.00 (6.652)	−2.386	0.017*
*PCRS* (positive)	2 months–1 year	Control	8	39.63 (7.009)	45.25 (3.370)	−2.207	0.027*
*PCRS* (negative)	Pretest–6 months	Placebo	12	12.50 (3.090)	15.00 (4.306)	−2.199	0.028*
*PCRS* (negative)	Posttest–1 year	Control	9	12.44 (3.167)	16.22 (3.801)	−2.033	0.042*

* *p* < 0.05.

As shown in Table 2, at baseline, the placebo group showed higher BAI and PCL‐5 than the others; BDI means were low and similar across groups. PCL‐5 was lowest in controls, moderate in the experimental group, and highest in placebo. PEDS means were higher in the experimental and placebo groups than in controls. PCRS‐Positive was high overall, whereas PCRS‐Negative peaked in the experimental group.

At 1 year, BAI and BDI decreased across all groups and remained low; PCL‐5 also declined. PEDS remained relatively higher in placebo, though between‐group differences were limited. PCRS‐Positive stayed high in all groups, while PCRS‐Negative peaked in controls.

Using pretest data, children's PEDS scores correlated positively with maternal anxiety (*r* = 0.659, *p* < 0.001), depression (*r* = 0.389, *p* = 0.019), and PTSD (PCL‐5; *r* = 0.668, *p* < 0.001). PCRS‐Positive correlated negatively with maternal anxiety (*r* = −0.442, *p* = 0.007) and depression (*r* = −0.446, *p* = 0.006). Full matrices are provided in Table S1.

At 1 year, PEDS correlated with maternal anxiety (*r* = 0.521, *p* = 0.002), depression (*r* = 0.729, *p* < 0.001), and PCL‐5 (*r* = 0.765, *p* < 0.001); FPC correlated with maternal depression (*r* = 0.491, *p* = 0.004) and PCL‐5 (*r* = 0.673, *p* < 0.001) (see Table S2).

As shown in Table 3, the model with maternal anxiety, depression and PCL‐5 was significant (*F*(3,32) = 10.310, *p* < 0.001). The model explained approximately 44% of the variance in PEDS scores (adjusted *R* ^2^ = 0.444). Maternal anxiety positively predicted PEDS (*β* = 0.486, *p* = 0.025); depression (*β* = −0.053, *p* = 0.739) and PCL‐5 (*β* = 0.290, *p* = 0.150) were not significant.

As shown in Table 4, the model was significant (*F*(3,32) = 2.924, *p* < 0.05). The model explained approximately 14% of the variance in the dependent variable (adjusted *R* ^2^ = 0.142). None of the individual predictors reached significance: anxiety (*β* = −0.310, *p* = 0.236), depression (*β* = −0.287, *p* = 0.151), PCL‐5 (*β* = 0.101, *p* = 0.680). The model was significant, yet no predictor showed a substantial unique effect on PCRS‐Positive.

As shown in Table 5, the model was significant (*F*(3,28) = 15,631, *p* < 0.001). The model explained approximately 59% of the variance in the dependent variable (adjusted *R* ^2^ = 0.586). PCL‐5 (*β* = 0.459, *p* = 0.073); anxiety (*β* = 0.109, *p* = 0.467), and depression (*β* = 0.284, *p* = 0.234) were non‐significant. The model was significant, yet no predictor showed a substantial unique effect on PEDS.

As shown in Table 6, the model was significant (*F*(3,28) = 10,048, *p* < 0.001). The model explained approximately 47% of the variance in the dependent variable (adjusted *R* ^2^ = 0.467). Maternal PCL‐5 significantly predicted FPC (*β* = 1.139, *p* < 0.001); anxiety (*β* = −0.128, *p* = 0.449) and depression (*β* = −0.441, *p* = 0.108) were not significant.

### 3.1 Between‐Group Comparisons

A significant difference was found only in the Positive Parent–Child Relationship scores, observed between the control and placebo groups, with the control group scoring higher (pretest *p* = 0.002; 1‐year follow‐up *p* = 0.048). No significant differences were found in the experimental group (*p* > 0.05).

### 3.2 Within‐Group Comparisons

We report within‐group changes across pre‐, post‐, 2‐, 6‐month and 1‐year follow‐ups; only significant results are shown here (full results are provided in the supplementary materials).

The MEACI was administered only to the experimental group at the first and final special play sessions. As shown in Table 7, scores decreased significantly for acceptance of the child (*z* = −3.071, *p* = 0.002), allowing the child to lead (*z* = −2.949, *p* = 0.003), involvement in play (*z* = −2.524, *p* = 0.012) and total empathy (*z* = −3.061, *p* = 0.002); lower scores indicate greater parental empathy (Güler Öztekin and Gülbahçe 2019).

As shown in Table 8, Loneliness/Sleep scores decreased in the experimental group at 2 months (*z* = −2.064, *p* = 0.039) and 1 year (*z* = −2.319, *p* = 0.020), and from 6 months to 1 year (*z* = −2.126, *p* = 0.033); in controls from pretest to 6 months (*z* = −2.414, *p* = 0.016) and 2 months to 1 year (*z* = −2.165, *p* = 0.030); and in placebo from pretest to 1 year (*z* = −2.200, *p* = 0.028) (see Table S3).

As shown in Table 9, impulsivity decreased in the experimental group from pretest to 1 year(*z* = −2.111, *p* = 0.035) and from 2 months to 1 year (*z* = −2.157, *p* = 0.031); in the placebo group from 2 to 6 months (*z* = −2.585, *p* = 0.010) and from 2 months to 1 year (*z* = −2.041, *p* = 0.041) (see Table S4).

As shown in Table 10, fear/anxiety rose in the experimental group from pretest to posttest and to 2‐, 6‐ and 12‐month follow‐ups (max *p* = 0.013). Controls showed the same pattern (max *p* = 0.011). In contrast, placebo scores decreased from posttest to 6 months (*z* = −2.136, *p* = 0.033) but rose again by 1 year (see Table S5).

As shown in Table 11, the experimental group's total stress showed a near‐significant drop at 2 months (*z* = −1.962, *p* = 0.051) and a significant reduction at 1 year (*z* = −2.485, *p* = 0.013); declines also continued from posttest to 1 year and from 6 months to 1 year. No change emerged in controls. In the placebo group, total stress decreased from pretest to 6 months (*z* = −2.268, *p* = 0.023), with additional reductions from pretest to 1 year and from posttest to 1 year. The experimental group showed sustained long‐term stress reductions, whereas the placebo group's decreases were less consistent (see Table S6).

As shown in Table 12, no significant changes were observed in the experimental group. In the control group, posttest→6‐month reductions emerged in Difficulty in Emotion Regulation (*z* = −2.13, *p* = 0.033), Insecurity and Negative Emotions (*z* = −2.21, *p* = 0.027), and FPC Total (*z* = −2.32, *p* = 0.020). In the placebo group, reductions from posttest to 1 year were found in Difficulty in Emotion Regulation (*z* = −2.09, *p* = 0.036), Insecurity and Negative Emotions (*z* = −2.07, *p* = 0.038) and FPC Total (*z* = −2.04, *p* = 0.041) (see Table S7).

As shown in Table 13, control group showed a decrease in PCRS‐Positive from pretest to 2 months (*z* = −2.386, *p* = 0.017) and an increase from 2 months to 1 year (*z* = −2.207, *p* = 0.027). PCRS‐Negative increased in controls from posttest to 1 year (*z* = −2.033, *p* = 0.042) and in placebo from pretest to 6 months (*z* = −2.199, *p* = 0.028). The experimental group showed no significant change on either PCRS subscale (*p*s > 0.05) (see Table S8).

## 4 Discussion

This study found that CPRT enhanced the empathy skills of mothers affected by the earthquake. Significant improvements were observed in the dimensions of accepting the child, allowing the child to lead, and involvement in play. This finding aligns with research conducted in Nepal reporting increased parental empathy following Filial Therapy post‐earthquake (Kim 2018). Similarly, studies on CPRT and Filial Therapy conducted across different cultural contexts have consistently demonstrated significant improvements in parental empathy (Bratton et al. 2011; Dillman Taylor et al. 2011; Güler Öztekin and Gülbahçe 2019).

PEDS total scores declined over time in both experimental and placebo groups. In the experimental arm, a near‐significant reduction emerged at 2 months (*p* = 0.051) and a significant one at 1 year, while the placebo group demonstrated significant decreases between 6 and 12 months. These parallel improvements indicate that the changes cannot be attributed solely to CPRT and do not demonstrate clear superiority over the placebo condition. It should also be considered that many changes in the placebo group may have stemmed from non‐specific processes. Such declines may reflect placebo effects (Sandler 2005), perceptual changes resulting from participation in structured interviews (Jones et al. 2017), caregiver rating shifts (Waschbusch et al. 2009), and natural recovery (Galatzer‐Levy et al. 2018; Usami et al. 2014). Within this context, the more consistent trajectory in the experimental group suggests that CPRT may have contributed as a supportive element, compatible with previous evidence showing that Filial Therapy reduces postearthquake PTSD, anxiety, somatization and suicide risk (Bahramian and Samsam Shariat 2024; Shen 2002; Kim 2018). Thus, total score patterns suggest that CPRT provides a partial and context‐dependent contribution to a multi‐factor recovery process.

In the PEDS Loneliness/Sleep subscale, improvements were observed at certain points across all groups. The control group showed a decrease beginning at the 6‐month follow‐up compared to the pretest, while the placebo group demonstrated a limited reduction at the 1‐year follow‐up. In contrast, the CPRT experimental group exhibited consistent decreases at both 2 months and 1 year, suggesting CPRT may have contributed to early and sustained effects in this domain. These patterns align with evidence linking stronger parent–child relationships to fewer sleep/loneliness problems (Cimon‐Paquet et al. 2019; Wang et al. 2021; Ahmadi Zadeh et al. 2022), with CPRT reducing separation anxiety (Ahmadi Zadeh et al. 2022) and normalizing clinical‐level sleep problems (Dillman Taylor et al. 2011). Overall, these findings support a partial therapeutic contribution of CPRT in the loneliness/sleep domain.

On the Impulsivity subscale, the experimental group demonstrated a significant reduction at the 1‐year follow‐up compared to the pretest (*p* = 0.035) and in the 2 months–1 year comparison (*p* = 0.031), indicating a sustained decline over time. In the placebo group, significant decreases were observed only in comparisons beginning from the 2‐month assessment (2–6 months, *p* = 0.010; 2 months–1 year, *p* = 0.041), while no significant change emerged in the control group. This pattern suggests that the trajectory of change in the experimental group differed from that of the placebo condition, although some later improvements were shared across groups.

Consistent with prior research demonstrating the role of parent‐focused play interventions in behavioural regulation (Shahrad et al. 2023; Sadr‐Salek et al. 2023), these findings indicate that CPRT may have contributed to reduced impulsivity through strengthening the parent–child relationship, within a broader context where non‐specific and contextual factors also played a role.

The emergence of PEDS total and effects not at posttest but at follow‐ups is defined in the literature as a ‘sleeper effect’ (van Aar et al. 2017). This pattern reflects the indirect nature of parent‐focused interventions: as mothers gradually apply newly learned skills, children's improvements may be delayed (Deković et al. 2010; Sofronoff et al. 2011). Effects may appear limited initially, then become clearer as parents internalize skills and children adapt (van Aar et al. 2017). Similarly, significant follow‐up reductions emerged in the COPEing with Toddler Behaviour study (Niccols 2009); and after 12 months in the Child FIRST intervention (Lowell et al. 2011). Thus, CPRT effects may be modest immediately posttreatment but become more evident and lasting over time.

Fear/anxiety increased at posttest, 2‐month, 6‐month and 1‐year assessments in both experimental and control groups. In the placebo group, the 6‐month decrease relative to posttest was not sustained; by 1 year, scores increased, as in the other groups. This likely reflects ongoing post‐earthquake adversity rather than CPRT ineffectiveness. Similarly, previous research has shown that posttraumatic fear, anxiety (Bozkurt Karalı and Özkan 2025) and trauma‐related symptoms (Forresi et al. 2020; Liberty et al. 2016; Parvaresh and Bahramnezhad 2009; Acharya et al. 2018; Silwal et al. 2022; Pynoos et al. 1993) may persist for years after trauma.

These findings align with the dose–response effect, whereby greater trauma exposure yields stronger psychological reactions (Pynoos et al. 1993). In our sample, children witnessed the earthquake and their parents' fear, endured aftershocks and inadequate living conditions and faced ongoing demolition with intrusive noise and dust. As Pynoos et al. (1993) noted, this condition further reinforced the persistence of traumatic memories and anxiety responses.

In the experimental group, no significant changes in FPC were observed at any measurement point, whereas significant improvements emerged in the control and placebo groups at the 6‐month and 1‐year assessments. The limited improvements observed in the control and placebo conditions may largely reflect natural recovery, as they emerged 18–24 months after the earthquake (Galatzer‐Levy et al. 2018). The absence of significant change in the experimental group may relate to maternal psychological burden, ongoing environmental adversities, small sample size and challenges of working with very young children. Consistent with our findings, several studies report no significant change in children's emotional/behavioural problems (Sergeant 2011; Brandt 1999; Hanif and Gul 2023). Nevertheless, substantial literature indicates that CPRT reduces internalizing and externalizing problems (Bratton et al. 2011; Carnes‐Holt 2010; Güler Öztekin and Gülbahçe 2019), suggesting that treatment response may vary by developmental stage, parental distress and contextual conditions.

Although no significant within‐group change in PCRS emerged for the experimental group, scores trended positive, whereas control and placebo worsened—suggesting a protective effect against relational deterioration. A ceiling effect—from initially high ratings (Brandt 1999)—and heightened maternal awareness likely masked detectable change.

The rise in children's fear scores and non‐significant FPC/PCRS results are not due solely to therapeutic limits but relate to persistent post‐earthquake conditions and sample characteristics, as detailed below.

Firstly, maternal depression, anxiety and especially PTSD strongly shaped children's adjustment: mothers' symptoms predicted child PTSD at pretest (44%) and follow‐ups (59%), and predicted emotional–behavioural problems at 1 year (47%), consistent with links between parental psychopathology and child PTSD/anxiety/behaviour problems (Morris et al. 2012; Bozkurt Karalı and Özkan 2025; Chen et al. 2020; Glaus et al. 2021; Lambert et al. 2014). Additionally, mothers' psychological symptoms affected the parent–child relationship early on. Although the model explained 14% of the variance, the individual contributions of each variable were limited. The literature similarly reports that parental PTSD is associated with insecure attachment (van Ee et al. 2016), reduced parental sensitivity (Gondoli & Silverberg, 1997), weaker parent–child relationships (Lauterbach et al. 2007), increased parenting stress (Hartzell et al. 2022) and controlling parenting practices (Christie et al. 2019). Overall, mothers' psychological symptoms appear to shape children's PTSD, emotional–behavioural adjustment and parent–child dynamics.

Secondly, according to the Disaster and Emergency Management Authority (AFAD 2025), frequent moderate aftershocks in the region during the measurement periods may have delayed recovery for both children and parents. Recurrent aftershocks reinforce loss of control and prolong anxiety and PTSD (Collins 2022; Dorahy and Kannis‐Dymand 2012). Evidence from Christchurch and Nepal indicates that prolonged aftershocks sustain trauma symptoms (Fergusson et al. 2014; Acharya et al. 2018), likely hindering recovery. Additionally, most participants continued living in affected areas, and proximity to the epicentre is linked to greater symptom severity and higher PTSD among those who remain (Najarian et al. 2017; Forresi et al. 2020; Acharya et al. 2018; Groome and Soureti 2004).

Thirdly, despite maternal anxiety, depression and PTSD scores below clinical cutoffs, persistent symptoms may have biased mothers' ratings of their children. The literature indicates that a parent's mental health can skew the appraisal of a child's symptoms, leading to biased or overreported assessments (Bromet et al. 2000; Müller and Furniss 2013).

Filial Therapy has shown efficacy for school‐age children after earthquakes (Kim 2018; Bahramian and Samsam Shariat 2024). Our study, however, targeted preschoolers, whose regulation relies more on the mother–child bond and parental post‐disaster responses (Boer et al. 2009; Dogan‐Ates 2010), suggesting that developmental factors may underlie the differing results.

Although the experimental group size was sufficient for implementing the intervention, evidence from clinical research suggests that small samples may limit statistical sensitivity and reduce the likelihood of detecting significant effects, even when meaningful changes are present (Heppner et al. 1992; Cao et al. 2024). Accordingly, some non‐significant or near‐significant findings in the present study—particularly in outcomes based on caregiver ratings of children—may be partially attributable to limited statistical power rather than the absence of therapeutic effects. Overall, these findings highlight the complexity of family‐based trauma interventions in early childhood, where developmental, relational and contextual factors jointly shape therapeutic outcomes.

### 4.1 Limitations

We initially planned to exclude mothers scoring above clinical cutoffs, but time constraints and the longitudinal design reduced retention, making it difficult to recruit lower‐symptom participants. Given the persistence of maternal symptoms after the Kahramanmaraş earthquakes (Bozkurt Karalı and Özkan 2025; Gareayaghi et al. 2025; Urnek and Kara 2025; Ustaoğlu et al. 2025), this proved impracticable. We therefore used the available sample, acknowledging these recruitment constraints as a limitation. Because the study focused on the mother–child dyad, potential effects of other family members were not assessed. Another limitation is reliance on mothers' reports; direct child data or observations could provide a fuller view.

## 5 Result

CPRT was linked to meaningful improvements in maternal empathy and to reductions in children's loneliness/sleep problems, impulsivity and posttraumatic emotional distress over time.

Rises in children's fear/anxiety and nonsignificant FPC/PCRS results are unlikely to reflect therapy limits alone; frequent aftershocks, slow reconstruction and unstable living conditions may have prolonged anxiety in both parents and children. Parents' psychological symptoms may have also skewed child ratings, while measurement challenges with preschoolers and the small sample likely reduced power and limited detectable effects.

Taken together, the findings do not demonstrate clear superiority of CPRT over other groups, particularly the placebo condition; rather, they suggest that CPRT functions as a potentially supportive component within a multi‐determined recovery process shaped by parental mental health, developmental characteristics and ongoing post‐earthquake conditions.

Therefore, CPRT appears to hold promise as a post‐disaster psychosocial intervention. Sustained effectiveness is likely to benefit from addressing parents' mental health, involving fathers/other caregivers and testing the intervention in larger samples. Overall, CPRT represents a promising family‐based approach to posttrauma recovery.

## Funding

This study was supported by TÜBİTAK (Project No. 223K295) and derived from the first author's doctoral dissertation conducted under the supervision of the other authors.

## Ethics Statement

This study was approved by the Hacettepe University Clinical Research Ethics Committee (approval number: KA‐23039) on 27 November 2023.

## Consent

Before participation, all mothers were informed about the study's purpose, procedures, voluntary nature and confidentiality. Written informed consent was obtained from all participating mothers for themselves and on behalf of their children prior to data collection.

## Conflicts of Interest

The authors declare no conflicts of interest.

## Supporting information


**Table S1:** Table of Intercorrelations Among Pretest Scales.
**Table S2:** Table of Intercorrelations Among One‐Year Follow‐Up Scales.
**Table S3:** Wilcoxon Signed‐Rank Test Results for PEDS Subscale: Loneliness/Sleep.
**Table S4:** Wilcoxon Signed‐Rank Test Results for PEDS Subscale: Impulsivity.
**Table S5:** Wilcoxon Signed‐Rank Test Results for PEDS Subscale: Fear/Anxiety.
**Table S6:** Wilcoxon Signed‐Rank Test Results for PEDS Total Score.
**Table S7:** Wilcoxon Signed‐Rank Test Results for Filial Problem Checklist (Subscales and Total Score).
**Table S8:** Wilcoxon Signed‐Rank Test Results for Parent–Child Relationship (Positive and Negative Subscale).

## Data Availability

The data that support the findings of this study are available from the corresponding author upon reasonable request. The data are not publicly available due to privacy or ethical restrictions. Additionally, the dataset was created in Turkish, which may pose language‐related challenges for international researchers.
