# Supplementary material for: Child–Parent Relationship Therapy for Earthquake‐Affected Preschoolers: A 1‐Year Controlled Study
Source: Clin Psychol Psychother. 2026 Apr 10;33(2):e70264. doi: 10.1002/cpp.70264 (PMC13066918; doi:10.1002/cpp.70264)
Supplement: Supplementary file 1 — Table S1: Table of Intercorrelations Among Pretest Scales. Table S2: Table of Intercorrelations Among One‐Year Follow‐Up Scales. Table S3: Wilcoxon Signed‐Rank Test Results for PEDS Subscale: Loneliness/Sleep. Table S4: Wilcoxon Signed‐Rank Test Results for PEDS Subscale: Impulsivity. Table S5: Wilcoxon Signed‐Rank Test Results for PEDS Subscale: Fear/Anxiety. Table S6: Wilcoxon Signed‐Rank Test Results for PEDS Total Score. Table S7: Wilcoxon Signed‐Rank Test Results for Filial Problem Checklist (Subscales and Total Score). Table S8: Wilcoxon Signed‐Rank Test Results for Parent–Child Relationship (Positive and Negative Subscale). [file CPP-33-e70264-s001.docx]

Appendix Table 1. Table of Intercorrelations Among Pretest Scales

| Scales |  | 1 | 2 | 3 | 4 | 5 | 6 | 7 | |
| --- | --- | --- | --- | --- | --- | --- | --- | --- | --- |
| 1.BAÖ | r |  | ,608** | ,820** | ,659** | ,508** | -,442** | ,251 | |
|  | p |  | <,001 | <,001 | <,001 | ,002 | ,007 | ,139 | |
| 2.BDÖ | r |  |  | ,520** | ,389* | ,351* | -,446** | ,245 | |
|  | p |  |  | ,001 | ,019 | ,036 | ,006 | ,150 | |
| 3.PCL-5 | r |  |  |  | ,668** | ,464** | -,325 | ,147 | |
|  | p |  |  |  | <,001 | ,004 | ,053 | ,393 | |
| 4.PEDS | r |  |  |  |  | ,557** | -,457** | ,182 | |
|  | p |  |  |  |  | <,001 | ,005 | ,289 | |
| 5. FPKL | r |  |  |  |  |  | -,409* | ,354* | |
|  | p |  |  |  |  |  | ,013 | ,034 | |
| 6.EÇİÖ (Positive) | r |  |  |  |  |  |  | ,236 | |
|  | p |  |  |  |  |  |  | ,165 | |
| 7.EÇİÖ (Negative) | r |  |  |  |  |  |  |  | |
|  | p |  |  |  |  |  |  |  | |
| *Note.* BAI=Beck Anxiety Inventory; BDI=Beck Depression Inventory; PCL-5=PTSD Checklist for DSM-5; PEDS=Pediatric Emotional Distress Scale; FPC=Filial Problem Checklist; PCRS (Positive)=Parent–Child Relationship Scale – Positive Relationship subscale; PCRS (Negative)=Parent–Child Relationship Scale – Negative Relationship subscale. | | | | | | | | | |

***p<0,01 *p<0,05*

Appendix Table 2. Table of Intercorrelations Among One-Year Follow-Up Scales

| Scales |  | 1 | 2 | 3 | 4 | 5 | 6 | 7 | |
| --- | --- | --- | --- | --- | --- | --- | --- | --- | --- |
| 1.BAÖ | r |  | ,704** | ,721** | ,311 | ,521** | ,043 | ,276 | |
|  | p |  | <,001 | <,001 | ,083 | ,002 | ,817 | ,126 | |
| 2.BDÖ | r |  |  | ,904** | ,491** | ,729** | -,138 | ,212 | |
|  | p |  |  | <,001 | ,004 | <,001 | ,451 | ,244 | |
| 3.PCL-5 | r |  |  |  | ,673** | ,765** | -,094 | ,272 | |
|  | p |  |  |  | <,001 | <,001 | ,609 | ,132 | |
| 4.FPKL | r |  |  |  |  | ,798** | -,091 | ,334 | |
|  | p |  |  |  |  | <,001 | ,620 | ,061 | |
| 5.PEDS | r |  |  |  |  |  | -,042 | ,334 | |
|  | p |  |  |  |  |  | ,818 | ,062 | |
| 6.EÇİÖ (Positive) | r |  |  |  |  |  |  | ,439* | |
|  | p |  |  |  |  |  |  | ,012 | |
| 7.EÇİÖ (Negative) | r |  |  |  |  |  |  |  | |
|  | p |  |  |  |  |  |  |  | |
| *Note.* BAI=Beck Anxiety Inventory; BDI=Beck Depression Inventory; PCL-5=PTSD Checklist for DSM-5; PEDS=Pediatric Emotional Distress Scale; FPC=Filial Problem Checklist; PCRS (Positive)=Parent–Child Relationship Scale – Positive Relationship subscale; PCRS (Negative)=Parent–Child Relationship Scale – Negative Relationship subscale. | | | | | | | | |  |

**p< 0,01 *p< 0,05

Appendix Table 3. Wilcoxon Signed-Rank Test Results for PEDS Subscale: Loneliness/Sleep

| Time 1 -Time 2  Comparison | Group | n | Time 1 Mean (SD) | Time 2 Mean (SD) | z | p |
| --- | --- | --- | --- | --- | --- | --- |
| Pretest – Posttest | Experimental | 12 | 6.83 (2.04) | 6.58 (2.39) | -0.268 | .788 |
|  | Control | 9 | 6.09 (1.97) | 5.82 (1.40) | -0.570 | .569 |
|  | Placebo | 12 | 6.92 (2.64) | 6.33 (2.61) | -0.836 | .403 |
| Pretest – 2nd-Month | Experimental | 12 | 6.83 (2.04) | 5.50 (2.02) | -2.064 | .039* |
|  | Control | 9 | 6.56 (1.88) | 5.89 (2.47) | -0.862 | .389 |
|  | Placebo | 12 | 6.92 (2.64) | 6.17 (2.33) | -0.569 | .569 |
| Pretest – 6th-Month | Experimental | 12 | 6.83 (2.04) | 5.92 (1.44) | -1.268 | .205 |
|  | Control | 9 | 6.56 (1.88) | 5.22 (2.17) | -2.414 | .016* |
|  | Placebo | 12 | 6.92 (2.64) | 5.25 (2.05) | -1.793 | .073 |
| Pretest – 1-Year | Experimental | 12 | 6.83 (2.04) | 5.08 (1.44) | -2.319 | .020* |
|  | Control | 9 | 6.44 (2.00) | 4.67 (2.30) | -2.309 | .021* |
|  | Placebo | 11 | 7.18 (2.60) | 5.55 (1.81) | -2.200 | .028* |
| Posttest – 2nd-Month | Experimental | 12 | 6.58 (2.39) | 5.50 (2.02) | -1.801 | .072 |
|  | Control | 9 | 5.56 (1.42) | 5.89 (2.47) | -0.258 | .796 |
|  | Placebo | 12 | 6.33 (2.61) | 6.17 (2.33) | -0.060 | .952 |
| Posttest – 6th-Month | Experimental | 12 | 6.58 (2.39) | 5.92 (1.44) | -1.215 | .224 |
|  | Control | 9 | 5.56 (1.42) | 5.22 (2.17) | -0.744 | .457 |
|  | Placebo | 12 | 6.33 (2.61) | 5.25 (2.05) | -1.620 | .105 |
| Posttest – 1-Year | Experimental | 12 | 6.58 (2.39) | 5.08 (1.44) | -2.203 | .028* |
|  | Control | 9 | 5.67 (1.50) | 4.67 (2.29) | -1.278 | .201 |
|  | Placebo | 11 | 6.55 (2.62) | 5.55 (1.81) | -1.897 | .058 |
| 2nd-Month – 6th-Month | Experimental | 12 | 5.50 (2.02) | 5.92 (1.44) | -0.710 | .478 |
|  | Control | 8 | 5.89 (2.47) | 5.22 (2.17) | -0.960 | .337 |
|  | Placebo | 12 | 6.17 (2.33) | 5.25 (2.05) | -1.547 | .122 |
| 2nd-Month – 1-Year | Experimental | 12 | 5.50 (2.02) | 5.08 (1.44) | -0.540 | .589 |
|  | Control | 8 | 6.00 (2.61) | 4.88 (2.36) | -2.165 | .030* |
|  | Placebo | 11 | 6.18 (2.44) | 5.55 (1.81) | -1.413 | .158 |
| 6th-Month – 1-Year | Experimental | 12 | 5.92 (1.44) | 5.08 (1.44) | -2.126 | .033* |
|  | Control | 8 | 5.38 (2.26) | 4.88 (2.36) | -0.816 | .414 |
|  | Placebo | 11 | 5.36 (2.11) | 5.55 (1.81) | -0.520 | .603 |

**p<0.05*

Appendix Table 4. Wilcoxon Signed-Rank Test Results for PEDS Subscale: Impulsivity

| Time 1 -Time 2  Comparison | Group | n | Time 1 Mean (SD) | Time 2 Mean (SD) | z | p |
| --- | --- | --- | --- | --- | --- | --- |
| Pretest – Posttest | Experimental | 12 | 9.58 (3.03) | 9.17 (2.76) | -0.565 | .572 |
|  | Control | 11 | 7.27 (2.83) | 7.45 (1.70) | -0.282 | .778 |
|  | Placebo | 12 | 9.25 (2.99) | 8.00 (2.99) | -0.851 | .395 |
| Pretest – 2nd-Month | Experimental | 12 | 9.58 (3.03) | 8.92 (2.97) | -0.741 | .458 |
|  | Control | 9 | 7.67 (3.00) | 6.56 (2.35) | -1.491 | .136 |
|  | Placebo | 12 | 9.25 (2.99) | 8.75 (2.77) | -0.141 | .888 |
| Pretest – 6th-Month | Experimental | 12 | 9.58 (3.03) | 8.50 (2.84) | -1.359 | .174 |
|  | Control | 9 | 7.67 (3.00) | 6.89 (2.37) | -1.160 | .246 |
|  | Placebo | 12 | 9.25 (2.99) | 7.42 (2.50) | -1.906 | .057 |
| Pretest – 1-Year | Experimental | 12 | 9.58 (3.03) | 8.00 (2.76) | -2.111 | . 035* |
|  | Control | 9 | 7.56 (3.00) | 7.00 (2.15) | -.085 | .396 |
|  | Placebo | 11 | 9.36 (3.11) | 7.73 (2.72) | -1.590 | .112 |
| Posttest – 2nd-Month | Experimental | 12 | 9.17 (2.76) | 8.92 (2.97) | -0.517 | .605 |
|  | Control | 9 | 7.00 (1.32) | 6.56 (2.35) | -1.081 | .279 |
|  | Placebo | 12 | 8.00 (2.99) | 8.75 (2.77) | -1.469 | .142 |
| Posttest – 6th-Month | Experimental | 12 | 9.17 (2.76) | 8.50 (2.84) | -1.178 | .239 |
|  | Control | 9 | 7.00 (1.32) | 6.89 (2.37) | -0.214 | .831 |
|  | Placebo | 12 | 8.00 (2.99) | 7.42 (2.50) | -1.643 | .100 |
| Posttest – 1-Year | Experimental | 12 | 9.17 (2.76) | 8.00 (2.76) | -1.919 | .055 |
|  | Control | 9 | 7.56(1.81) | 6.89 (2.15) | -1.200 | .230 |
|  | Placebo | 11 | 8.09 (3.11) | 7.73 (2.72) | -0.921 | .357 |
| 2nd-Month – 6th-Month | Experimental | 12 | 8.92 (2.97) | 8.50 (2.84) | -1.186 | .236 |
|  | Control | 9 | 6.56 (2.35) | 6.89 (2.37) | -0.108 | .914 |
|  | Placebo | 12 | 8.75 (2.77) | 7.42 (2.50) | -2.585 | .010* |
| 2nd-Month – 1-Year | Experimental | 12 | 8.92 (2.97) | 8.00 (2.76) | -2.157 | .031* |
|  | Control | 8 | 6.63 (2.50) | 6.75 (2.25) | -0.141 | .888 |
|  | Placebo | 11 | 8.82 (2.89) | 7.73 (2.72) | -2.041 | .041* |
| 6th-Month – 1-Year | Experimental | 12 | 8.50 (2.84) | 8.00 (2.76) | -1.897 | .058 |
|  | Control | 8 | 7.13 (2.42) | 6.75 (2.25) | -0.414 | .679 |
|  | Placebo | 11 | 7.64 (2.50) | 7.73 (2.72) | -0.213 | .832 |

**p<0.05*

Appendix Table 5. Wilcoxon Signed-Rank Test Results for PEDS Subscale: Fear/Anxiety

| Time 1 -Time 2  Comparison | Group | n | Time 1 Mean (SD) | Time 2 Mean (SD) | z | p |
| --- | --- | --- | --- | --- | --- | --- |
| Pretest – Posttest | Experimental | 12 | 3.00 (1.04) | 3.92 (1.17) | -2.495 | .013* |
|  | Control | 11 | 2.73 (1.01) | 4.91 (1.70) | -2.961 | .003* |
|  | Placebo | 12 | 3.92 (2.15) | 4.50 (1.31) | -1.269 | .204 |
| Pretest – 2nd-Month | Experimental | 12 | 3.00 (1.04) | 4.08 (1.62) | -2.588 | .010* |
|  | Control | 9 | 2.78 (1.09) | 4.78 (2.17) | -2.555 | .011* |
|  | Placebo | 12 | 3.92 (2.15) | 4.50 (1.88) | -0.857 | .391 |
| Pretest – 6th-Month | Experimental | 12 | 3.00 (1.04) | 4.25 (1.42) | -2.683 | .007* |
|  | Control | 9 | 2.78 (1.09) | 4.44 (2.13) | -2.807 | .005* |
|  | Placebo | 12 | 3.92 (2.15) | 3.50 (0.67) | -0.329 | .742 |
| Pretest – 1-Year | Experimental | 12 | 3.00 (1.04) | 3.75 (1.06) | -2.714 | .007* |
|  | Control | 9 | 2.78 (1.09) | 4.78 (1.87) | -2.699 | .007* |
|  | Placebo | 11 | 4.09 (2.17) | 4.73 (1.27) | -1.282 | .200 |
| Posttest – 2nd-Month | Experimental | 12 | 3.92 (1.17) | 4.08 (1.62) | -0.707 | .480 |
|  | Control | 9 | 5.00 (1.87) | 4.78 (2.17) | -0.324 | .746 |
|  | Placebo | 12 | 4.50 (1.31) | 4.50 (1.88) | -0.367 | .713 |
| Posttest – 6th-Month | Experimental | 12 | 3.92 (1.17) | 4.25 (1.42) | -1.633 | .102 |
|  | Control | 9 | 5.00 (1.87) | 4.44 (2.13) | -1.518 | .129 |
|  | Placebo | 12 | 4.50 (1.31) | 3.50 (0.67) | -2.136 | .033* |
| Posttest – 1-Year | Experimental | 12 | 3.92 (1.17) | 3.75 (1.06) | -0.707 | .480 |
|  | Control | 9 | 4.89 (1.90) | 4.78 (2.17) | -0.832 | .405 |
|  | Placebo | 11 | 4.64 (1.29) | 4.73 (1.27) | -0.447 | .655 |
| 2nd-Month – 6th-Month | Experimental | 12 | 4.08 (1.62) | 4.25 (1.42) | -0.707 | .480 |
|  | Control | 9 | 4.78 (2.17) | 4.44 (2.13) | -0.966 | .334 |
|  | Placebo | 12 | 4.50 (1.88) | 3.50 (0.67) | -1.437 | .151 |
| 2nd-Month – 1-Year | Experimental | 12 | 4.08 (1.62) | 3.75 (1.06) | -1.000 | .317 |
|  | Control | 8 | 4.75 (2.32) | 5.00 (1.85) | -0.707 | .480 |
|  | Placebo | 11 | 4.55 (1.97) | 4.73 (1.27) | -0.742 | .458 |
| 6th-Month – 1-Year | Experimental | 12 | 4.25 (1.42) | 3.75 (1.06) | -1.540 | .124 |
|  | Control | 8 | 4.63 (2.20) | 5.00 (1.85) | -1.134 | .257 |
|  | Placebo | 11 | 3.55 (0.69) | 4.73 (1.27) | -2.232 | .026* |

**p<0.05*

Appendix Table 6. Wilcoxon Signed-Rank Test Results for PEDS Total Score

| Time 1 -Time 2  Comparison | Group | n | Time 1  Mean (SD) | Time 2  Mean (SD) | z | p |
| --- | --- | --- | --- | --- | --- | --- |
| Pretest – Posttest | Experimental | 12 | 26.92 (7.62) | 24.58 (5.90) | -1.020 | .308 |
|  | Control | 11 | 22.45 (6.79) | 23.18 (4.22) | -0.566 | .572 |
|  | Placebo | 12 | 29.25 (10.63) | 24.17 (6.24) | -1.686 | .092 |
| Pretest – 2nd-Month | Experimental | 12 | 26.92 (7.62) | 23.67 (7.17) | -1.962 | .051 |
|  | Control | 9 | 23.67 (6.95) | 22.44 (7.09) | -1.124 | .261 |
|  | Placebo | 12 | 29.25 (10.63) | 24.83 (6.86) | -1.126 | .260 |
| Pretest – 6th-Month | Experimental | 12 | 26.92 (7.62) | 24.08 (5.99) | -1.735 | .083 |
|  | Control | 9 | 23.67 (6.95) | 21.89 (7.54) | -1.362 | .173 |
|  | Placebo | 12 | 29.25 (10.63) | 21.67 (5.09) | -2.268 | .023* |
| Pretest – 1-Year | Experimental | 12 | 26.92 (7.62) | 22.17 (5.92) | -2.485 | .013* |
|  | Control | 9 | 23.44 (7.18) | 21.44 (6.23) | -0.894 | .371 |
|  | Placebo | 11 | 29.91 (10.89) | 23.27 (5.82) | -2.100 | .036* |
| Posttest – 2nd-Month | Experimental | 12 | 24.58 (5.90) | 23.67 (7.17) | -0.625 | .532 |
|  | Control | 9 | 22.67 (4.47) | 22.44 (7.09) | -0.922 | .357 |
|  | Placebo | 12 | 24.17 (6.24) | 24.83 (6.86) | -0.563 | .574 |
| Posttest – 6th-Month | Experimental | 12 | 24.58 (5.90) | 24.08 (5.99) | -0.492 | .623 |
|  | Control | 9 | 22.67 (4.47) | 21.89 (7.54) | -0.914 | .361 |
|  | Placebo | 12 | 24.17 (6.24) | 21.67 (5.09) | -1.891 | .059 |
| Posttest – 1-Year | Experimental | 12 | 24.58 (5.90) | 22.17 (5.92) | -2.011 | .044* |
|  | Control | 9 | 22.67 (4.47) | 21.44 (6.23) | -1.129 | .259 |
|  | Placebo | 11 | 24.64 (6.31) | 23.27 (5.82) | -1.973 | .049* |
| 2nd-Month – 6th-Month | Experimental | 12 | 23.67 (7.17) | 24.08 (5.99) | -0.898 | .369 |
|  | Control | 9 | 22.44 (7.09) | 21.89 (7.54) | -0.597 | .550 |
|  | Placebo | 12 | 24.83 (6.86) | 21.67 (5.09) | -1.633 | .102 |
| 2nd-Month – 1-Year | Experimental | 12 | 23.67 (7.17) | 22.17 (5.92) | -1.495 | .135 |
|  | Control | 8 | 22.75 (7.52) | 21.50 (6.65) | -1.279 | .201 |
|  | Placebo | 11 | 25.00 (7.17) | 23.27 (5.82) | -1.011 | .312 |
| 6th-Month – 1-Year | Experimental | 12 | 24.08 (5.99) | 22.17 (5.92) | -2.469 | .014* |
|  | Control | 8 | 22.50 (7.82) | 21.50 (6.65) | -0.512 | .609 |
|  | Placebo | 11 | 22.18 (5.00) | 23.27 (5.82) | -0.846 | .397 |

**p<0.05*

Appendix Table 7. Wilcoxon Signed-Rank Test Results for Filial Problem Checklist (Subscales and Total Score)

| Subscale | Time 1 -Time 2  Comparison | Group | n | Time 1  Mean (SD) | Time 2  Mean (SD) | z | p |
| --- | --- | --- | --- | --- | --- | --- | --- |
| Emotional Control Difficulties | Pretest – Posttest | Experimental | 12 | 19,50 (14,538) | 13,67 (13,398) | -1,729 | ,084 |
|  |  | Control | 11 | 12,27 (16,390) | 12,73 (16,438) | -,089 | ,929 |
|  |  | Placebo | 12 | 17,08 (18,238) | 20,17 (12,305) | -,801 | ,423 |
|  | Pretest – 2nd-Month | Experimental | 12 | 19,50 (14,538) | 19,67 (14,234) | -,178 | ,858 |
|  |  | Control | 9 | 12,89 (18,052) | 15,67 (18,062) | -,071 | ,943 |
|  |  | Placebo | 12 | 17,08 (18,238) | 20,67 (14,687) | -,561 | ,575 |
|  | Pretest – 6th-Month | Experimental | 12 | 19,50 (14,538) | 16,58 (16,256) | -,935 | ,350 |
|  |  | Control | 9 | 12,89 (18,052) | 11,22 (17,541) | -1,424 | ,154 |
|  |  | Placebo | 12 | 17,08 (18,238) | 17,58 (13,324) | -,044 | ,965 |
|  | Pretest – 1-Year | Experimental | 12 | 19,50 (14,538) | 17,58 (16,572) | -1,109 | ,268 |
|  |  | Control | 9 | 14,44  (17,494) | 12,78  (13,609) | -,841 | ,400 |
|  |  | Placebo | 11 | 17,09 (19,128) | 14,09 (12,942) | -,830 | ,407 |
|  | Posttest – 2nd-Month | Experimental | 12 | 13,67  (13,398) | 19,67  (14,234) | -1,379 | ,168 |
|  |  | Control | 9 | 13,67  (17,727) | 15,67  (18,062) | -,297 | ,766 |
|  |  | Placebo | 12 | 20,17  (12,305) | 20,67  (14,687) | -,044 | ,965 |
|  | Posttest – 6th-Month | Experimental | 12 | 13,67 (13,398) | 16,58 (16,256) | -,865 | ,387 |
|  |  | Control | 9 | 13,67 (17,727) | 11,22 (17,541) | -2,132 | ,033* |
|  |  | Placebo | 12 | 20,17 (12,305) | 17,58 (13,324) | -1,290 | ,197 |
|  | Posttest – 1-Year | Experimental | 12 | 13,67 (13,398) | 17,58 (16,572) | -,668 | ,504 |
|  |  | Control | 9 | 15,56 (16,979) | 12,78 (13,609) | -,847 | ,397 |
|  |  | Placebo | 11 | 20,00 (12,892) | 14,09 (12,942) | -2,092 | ,036* |
|  | 2nd-Month – 6th-Month | Experimental | 12 | 19,67 (14,234) | 16,58 (16,256) | -1,112 | ,266 |
|  |  | Control | 9 | 15,67 (18,062) | 11,22 (17,541) | -1,628 | ,103 |
|  |  | Placebo | 12 | 20,67 (14,687) | 17,58 (13,324) | -,713 | ,476 |
|  | 2nd-Month – 1-Year | Experimental | 12 | 19,67 (14,234) | 17,58 (16,572) | -1,419 | ,156 |
|  |  | Control | 8 | 17,25 (18,630) | 10,75 (13,014) | -1,355 | ,176 |
|  |  | Placebo | 11 | 21,82 (14,824) | 14,09 (12,942) | -1,836 | ,066 |
|  | 6th-Month – 1-Year | Experimental | 12 | 16,58 (16,256) | 17,58 (16,572) | -,433 | ,665 |
|  |  | Control | 8 | 12,63 (18,205) | 10,75 (13,014) | -,593 | ,553 |
|  |  | Placebo | 11 | 18,82 (13,235) | 14,09 (12,942) | -1,684 | ,092 |
| Filial Problem Checklist Total | Pretest – Posttest | Experimental | 12 | 47,67 (36,212) | 34,67 (36,619) | -1,423 | ,155 |
|  |  | Control | 11 | 36,18 (46,447) | 34,09 (43,351) | -,712 | ,477 |
|  |  | Placebo | 12 | 53,08 (77,495) | 48,75 (30,094) | -,622 | ,534 |
|  | Pretest – 2nd-Month | Experimental | 12 | 47,67 (36,212) | 48,33 (42,630) | -,045 | ,964 |
|  |  | Control | 9 | 39,11 (50,866) | 39,78 (44,409) | -1,126 | ,260 |
|  |  | Placebo | 12 | 53,08 (77,495) | 61,25 (68,771) | -,311 | ,756 |
|  | Pretest – 6th-Month | Experimental | 12 | 47,67 (36,212) | 40,33 (41,764) | -1,257 | ,209 |
|  |  | Control | 9 | 39,11 (50,866) | 30,44 (45,092) | -1,601 | ,109 |
|  |  | Placebo | 12 | 53,08 (77,495) | 52,75 (66,865) | -,267 | ,790 |
|  | Pretest – 1-Year | Experimental | 12 | 47,67 (36,212) | 45,67 (43,866) | -1,138 | ,255 |
|  |  | Control | 9 | 42,67  (49,358) | 29,67  (35,405) | -1,540 | ,123 |
|  |  | Placebo | 11 | 54,18 (81,180) | 36,91 (37,774) | -,968 | ,333 |
|  | Posttest – 2nd-Month | Experimental | 12 | 34,67  (36,619) | 48,33  (42,630) | -1,256 | 209 |
|  |  | Control | 9 | 36,89  (46,745) | 39,78  (44,409) | -,889 | ,374 |
|  |  | Placebo | 12 | 48,75  (30,094) | 61,25  (68,771) | -.356 | ,722 |
|  | Posttest – 6th-Month | Experimental | 12 | 34,67 (36,619) | 40,33 (41,764) | -,267 | ,790 |
|  |  | Control | 9 | 36,89 (46,745) | 30,44 (45,092) | -2,325 | ,020* |
|  |  | Placebo | 12 | 48,75 (30,094) | 52,75 (66,865) | -,622 | ,534 |
|  | Posttest – 1-Year | Experimental | 12 | 34,67 (36,619) | 45,67 (43,866) | -,393 | ,695 |
|  |  | Control | 9 | 41,33 (44,989) | 29,67 (35,405) | -1,246 | ,213 |
|  |  | Placebo | 11 | 48,73 (31,563) | 36,91 (37,774) | -2,040 | ,041* |
|  | 2nd-Month – 6th-Month | Experimental | 12 | 48,33 (42,630) | 40,33 (41,764) | -,864 | ,388 |
|  |  | Control | 9 | 39,78 (44,409) | 30,44 (45,092) | -1,129 | ,259 |
|  |  | Placebo | 12 | 61,25 (68,771) | 52,75 (66,865) | -,445 | ,656 |
|  | 2nd-Month – 1-Year | Experimental | 12 | 48,33 (42,630) | 45,67 (43,866) | -,902 | ,367 |
|  |  | Control | 8 | 44,13 (45,382) | 25,00 (34,765) | -1,820 | ,069 |
|  |  | Placebo | 11 | 65,64 (70,345) | 36,91 (37,774) | -1,718 | ,086 |
|  | 6th-Month – 1-Year | Experimental | 12 | 40,33 (41,764) | 45,67 (43,866) | -,713 | ,476 |
|  |  | Control | 8 | 34,25 (46,635) | 25,00 (34,765) | -1,120 | ,263 |
|  |  | Placebo | 11 | 56,55 (68,760) | 36,91 (37,774) | -1,480 | ,139 |
| Insecurity and Negative Emotions | Pretest – Posttest | Experimental | 12 | 12,50 (5,713) | 8,75 (8,170) | -1,729 | ,084 |
|  |  | Control | 11 | 12,73 (13,192) | 11,18 (14,176) | -1,385 | ,166 |
|  |  | Placebo | 12 | 14,33 (16,283) | 13,42 (9,482) | -,356 | ,722 |
|  | Pretest – 2nd-Month | Experimental | 12 | 12,50 (5,713) | 11,50 (10,068) | -1,250 | ,211 |
|  |  | Control | 9 | 14,22 (14,131) | 12,11 (13,706) | -1,429 | ,153 |
|  |  | Placebo | 12 | 14,33 (16,283) | 14,58 (16,003) | -,133 | ,894 |
|  | Pretest – 6th-Month | Experimental | 12 | 12,50 (5,713) | 9,58 (8,096) | -1,415 | ,157 |
|  |  | Control | 9 | 14,22 (14,131) | 10,67 (14,177) | -2,032 | ,042* |
|  |  | Placebo | 12 | 14,33 (16,283) | 12,58 (15,389) | -,716 | ,474 |
|  | Pretest – 1-Year | Experimental | 12 | 12,50 (5,713) | 11,33 (9,930) | -,824 | ,410 |
|  |  | Control | 9 | 14,78  (13,818) | 8,11  (10,948) | -2,524 | ,012* |
|  |  | Placebo | 11 | 14,00 (17,035) | 9,45 (8,583) | -1,277 | ,201 |
|  | Posttest – 2nd-Month | Experimental | 12 | 8,75  (8,170) | 11,50  (10,068) | -,631 | ,528 |
|  |  | Control | 9 | 13,00  (15,116) | 12,11  (13,706) | -1,193 | ,233 |
|  |  | Placebo | 12 | 13,42  (9,482) | 14,58  (16,003) | -,255 | ,799 |
|  | Posttest – 6th-Month | Experimental | 12 | 8,75 (8,170) | 9,58 (8,096) | -,492 | ,622 |
|  |  | Control | 9 | 13,00 (15,116) | 10,67 (14,177) | -2,214 | ,027* |
|  |  | Placebo | 12 | 13,42 (9,482) | 12,58 (15,389) | -,624 | ,533 |
|  | Posttest – 1-Year | Experimental | 12 | 8,75 (8,170) | 11,33 (9,930) | -,433 | ,665 |
|  |  | Control | 9 | 13,33 (14,900) | 8,11 (10,948) | -1,844 | ,065 |
|  |  | Placebo | 11 | 13,18 (9,908) | 9,45 (8,583) | -2,077 | ,038* |
|  | 2nd-Month – 6th-Month | Experimental | 12 | 11,50 (10,068) | 9,58 (8,096) | -,447 | ,655 |
|  |  | Control | 9 | 12,11 (13,706) | 10,67 (14,177) | -1,005 | ,315 |
|  |  | Placebo | 12 | 14,58 (16,003) | 12,58 (15,389) | -,664 | ,507 |
|  | 2nd-Month – 1-Year | Experimental | 12 | 11,50 (10,068) | 11,33 (9,930) | -,668 | ,504 |
|  |  | Control | 8 | 13,38 (14,081) | 8,63 (11,587) | -1,544 | ,123 |
|  |  | Placebo | 11 | 15,55 (16,416) | 9,45 (8,583) | -1,429 | ,153 |
|  | 6th-Month – 1-Year | Experimental | 12 | 9,58 (8,096) | 11,33 (9,930) | -,308 | ,758 |
|  |  | Control | 8 | 12,00 (14,541) | 8,63 (11,587) | -1,474 | ,141 |
|  |  | Placebo | 11 | 13,27 (15,944) | 9,45 (8,583) | -,869 | ,385 |
| Problematic Social Behaviors | Pretest – Posttest | Experimental | 12 | 6,50 (7,693) | 6,08 (8,140) | ,000 | 1,000 |
|  |  | Control | 11 | 4,00 (5,831) | 3,91 (5,394) | -,171 | ,865 |
|  |  | Placebo | 12 | 10,08 (21,853) | 6,42 (6,667) | -,089 | ,929 |
|  | Pretest – 2nd-Month | Experimental | 12 | 6,50 (7,693) | 8,50 (8,837) | -1,663 | ,096 |
|  |  | Control | 9 | 4,00 (6,205) | 4,00 (4,500) | ,000 | 1,000 |
|  |  | Placebo | 12 | 10,08 (21,853) | 13,17 (20,036) | -,306 | ,759 |
|  | Pretest – 6th-Month | Experimental | 12 | 6,50 (7,693) | 6,50 (9,100) | -,119 | ,906 |
|  |  | Control | 9 | 4,00 (6,205) | 2,67 (4,899) | -2,232 | ,026* |
|  |  | Placebo | 12 | 10,08 (21,853) | 10,00 (19,470) | -,297 | ,767 |
|  | Pretest – 1-Year | Experimental | 12 | 6,50 (7,693) | 7,75 (9,771) | -,281 | ,779 |
|  |  | Control | 9 | 4,89  (6,133) | 1,78  (3,153) | -2,536 | ,011* |
|  |  | Placebo | 11 | 10,82 (22,763) | 6,64 (8,857) | -,358 | ,721 |
|  | Posttest – 2nd-Month | Experimental | 12 | 6,08  (8,140) | 8,50  (8,837) | -1,190 | ,234 |
|  |  | Control | 9 | 3,56  (5,294) | 4,00  (4,500) | ,000 | 1,000 |
|  |  | Placebo | 12 | 6,42  (6,667) | 13,17  (20,036) | -,995 | ,320 |
|  | Posttest – 6th-Month | Experimental | 12 | 6,08 (8,140) | 6,50 (9,100) | -,070 | ,944 |
|  |  | Control | 9 | 3,56 (5,294) | 2,67 (4,899) | -1,890 | ,059 |
|  |  | Placebo | 12 | 6,42 (6,667) | 10,00 (19,470) | -,071 | ,943 |
|  | Posttest – 1-Year | Experimental | 12 | 6,08 (8,140) | 7,75 (9,771) | -,051 | ,959 |
|  |  | Control | 9 | 4,78 (5,630) | 1,78 (3,153) | -1,997 | ,046* |
|  |  | Placebo | 11 | 6,91 (6,760) | 6,64 (8,857) | ,000 | 1,000 |
|  | 2nd-Month – 6th-Month | Experimental | 12 | 8,50 (8,837) | 6,50 (9,100) | -,771 | ,440 |
|  |  | Control | 9 | 4,00 (4,500) | 2,67 (4,899) | -,763 | ,445 |
|  |  | Placebo | 12 | 13,17 (20,036) | 10,00 (19,470) | -1,548 | ,122 |
|  | 2nd-Month – 1-Year | Experimental | 12 | 8,50 (8,837) | 7,75 (9,771) | -,831 | ,406 |
|  |  | Control | 8 | 4,50 (4,536) | 1,75 (3,370) | -2,226 | ,026* |
|  |  | Placebo | 11 | 14,36 (20,559) | 6,64 (8,857) | -1,660 | ,097 |
|  | 6th-Month – 1-Year | Experimental | 12 | 6,50 (9,100) | 7,75 (9,771) | -,492 | ,623 |
|  |  | Control | 8 | 3,00 (5,127) | 1,75 (3,370) | -1,300 | ,194 |
|  |  | Placebo | 11 | 10,91 (20,152) | 6,64 (8,857) | -,924 | ,355 |

**p<0.05*

Appendix Table 8. Wilcoxon Signed-Rank Test Results for Parent–Child Relationship (Positive and Negative Subscale)

| Subscale | Time1-Time2 Comparison | Group | n | Time 1  Mean (SD) | Time 2  Mean (SD) | z | p |
| --- | --- | --- | --- | --- | --- | --- | --- |
| PCRS (Positive) | Pretest – Posttest | Experimental | 12 | 41,92 (3,65) | 42,67 (2,38) | -,632 | ,528 |
|  |  | Control | 11 | 45,45 (2,65) | 41,91 (6,30) | -1,889 | ,059 |
|  |  | Placebo | 12 | 38,58 (5,28) | 40,00 (4,80) | -,894 | ,371 |
|  | Pretest – 2nd-Month | Experimental | 12 | 41,92 (3,65) | 40,67 (4,67) | -1,381 | ,167 |
|  |  | Control | 9 | 45,22 (2,81) | 40,00 (6,65) | -2,386 | ,017* |
|  |  | Placebo | 12 | 38,58 (5,28) | 39,92 (6,68) | -,756 | ,450 |
|  | Pretest – 6th-Month | Experimental | 12 | 41,92 (3,65) | 41,50 (4,05) | -,317 | ,751 |
|  |  | Control | 9 | 45,22 (2,81) | 43,56 (5,07) | -1,368 | ,171 |
|  |  | Placebo | 12 | 38,58 (5,28) | 39,92 (4,92) | -,716 | ,474 |
|  | Pretest – 1-Year | Experimental | 12 | 41,92 (3,65) | 42,25 (5,01) | -,039 | ,969 |
|  |  | Control | 9 | 45,44 (2,96) | 44,67 (3,60) | -431 | ,667 |
|  |  | Placebo | 11 | 38,09 (5,24) | 37,09 (10,38) | -,422 | ,673 |
|  | Posttest – 2nd-Month | Experimental | 12 | 42,67 (2,38) | 40,67 (4,67) | -1,404 | ,160 |
|  |  | Control | 9 | 43,67 (4,63) | 40,00 (6,65) | -1,014 | ,311 |
|  |  | Placebo | 12 | 40,00 (4,80) | 39,92 (6,68) | -267 | ,789 |
|  | Posttest – 6th-Month | Experimental | 12 | 42,67 (2,38) | 41,50 (4,05) | -1,350 | ,177 |
|  |  | Control | 9 | 43,67 (4,63) | 43,56 (5,07) | -,421 | ,674 |
|  |  | Placebo | 12 | 40,00 (4,80) | 39,92 (4,92) | -,051 | ,959 |
|  | Posttest – 1-Year | Experimental | 12 | 42,67 (2,38) | 42,25 (5,01) | -,103 | ,918 |
|  |  | Control | 9 | 42,44 (6,912) | 44,67 (3,606) | -1,014 | ,310 |
|  |  | Placebo | 11 | 40,09 (5,02) | 37,09 (10,38) | -,805 | ,421 |
|  | 2nd-Month – 6th-Month | Experimental | 12 | 40,67 (4,67) | 41,50 (4,05) | -,492 | ,623 |
|  |  | Control | 9 | 40,00 (6,65) | 43,56 (5,07) | -,771 | ,441 |
|  |  | Placebo | 12 | 39,92 (6,68) | 39,92 (4,92) | -,549 | ,583 |
|  | 2nd-Month – 1-Year | Experimental | 12 | 40,67 (4,67) | 42,25 (5,01) | -1,029 | ,304 |
|  |  | Control | 8 | 39,63 (7,00) | 45,25 (3,37) | -2,207 | ,027* |
|  |  | Placebo | 11 | 40,18 (6,94) | 37,09 (10,38) | -1,605 | ,108 |
|  | 6th-Month – 1-Year | Experimental | 12 | 41,50 (4,05) | 42,25 (5,01) | -,747 | ,455 |
|  |  | Control | 8 | 43,00 (5,12) | 45,25 (3,37) | -1,866 | ,062 |
|  |  | Placebo | 11 | 40,09 (5,12) | 37,09 (10,38) | -,564 | ,573 |
| PCRS (Negative) | Pretest – Posttest | Experimental | 12 | 15,33 (4,88) | 14,67 (4,57) | -,758 | ,448 |
|  |  | Control | 11 | 14,09 (3,44) | 12,36 (2,97) | -1,181 | ,238 |
|  |  | Placebo | 12 | 12,50 (3,090) | 14,08 (2,021) | -1,383 | ,167 |
|  | Pretest – 2nd-Month | Experimental | 12 | 15,33 (4,88) | 14,92 (4,73) | -,357 | ,721 |
|  |  | Control | 9 | 14,33 (3,24) | 13,11 (3,65) | -,561 | ,575 |
|  |  | Placebo | 12 | 12,50 (3,09) | 13,83 (2,98) | -,893 | ,372 |
|  | Pretest – 6th-Month | Experimental | 12 | 15,33 (4,88) | 14,17 (4,56) | -1,387 | ,165 |
|  |  | Control | 9 | 14,33 (3,24) | 14,33 (2,12) | -,344 | ,731 |
|  |  | Placebo | 12 | 12,50 (3,09) | 15,00 (4,30) | -2,199 | ,028* |
|  | Pretest – 1-Year | Experimental | 12 | 15,33 (4,88) | 14,67 (5,08) | -,866 | ,386 |
|  |  | Control | 9 | 15,11 (2,89) | 16,22 (3,80) | -772 | ,440 |
|  |  | Placebo | 11 | 12,18 (3,02) | 13,18 (4,33) | -,297 | ,766 |
|  | Posttest – 2nd-Month | Experimental | 12 | 42,67 (2,38) | 40,67 (4,67) | -1,40 | 160 |
|  |  | Control | 9 | 12,22 (3,27) | 13,11 (3,65) | -595 | ,552 |
|  |  | Placebo | 12 | 14,08 (2,02) | 13,83 (2,98) | -,404 | ,686 |
|  | Posttest – 6th-Month | Experimental | 12 | 14,67 (4,57) | 14,17 (4,56) | -,312 | ,755 |
|  |  | Control | 9 | 12,22 (3,27) | 14,33 (2,12) | -1,368 | ,171 |
|  |  | Placebo | 12 | 14,08 (2,02) | 15,00 (4,30) | -,631 | ,528 |
|  | Posttest – 1-Year | Experimental | 12 | 14,67 (4,57) | 14,67 (5,08) | -,140 | ,888 |
|  |  | Control | 9 | 12,44 (3,16) | 16,22 (3,80) | -2,033 | ,042* |
|  |  | Placebo | 11 | 14,00 (2,09) | 13,18 (4,33) | -,773 | ,440 |
|  | 2nd-Month – 6th-Month | Experimental | 12 | 14,92 (4,73) | 14,17 (4,56) | -,717 | ,474 |
|  |  | Control | 9 | 13,11 (3,65) | 14,33 (2,12) | -,677 | ,498 |
|  |  | Placebo | 12 | 13,83 (2,98) | 15,00 (4,30) | -,551 | ,581 |
|  | 2nd-Month – 1-Year | Experimental | 12 | 14,92 (4,73) | 14,67 (5,08) | -,360 | ,719 |
|  |  | Control | 8 | 13,88 (3,04) | 16,25 (4,06) | -1,119 | ,263 |
|  |  | Placebo | 11 | 13,55 (2,94) | 13,18 (4,33) | -,085 | ,932 |
|  | 6th-Month – 1-Year | Experimental | 12 | 14,17 (4,56) | 14,67 (5,08) | -,257 | ,797 |
|  |  | Control | 8 | 14,50 (2,20) | 16,25 (4,06) | -1,490 | ,136 |
|  |  | Placebo | 11 | 15,00 (4,51) | 13,18 (4,33) | -1,188 | ,235 |

**p<0.05*
